# Eucalyptus Oil

**Published:** 1894-06-16

**Authors:** J. B. Curgenven

**Affiliations:** Teddington


### Editor's Letter-Box.

[Our correspondents are reminded that proxility is a great bar to publication, and that brevity of style and conciseness of statement greatly facilitate early insertion.]

### EUCALYPTUS OIL.

Sir,—The case of eucalyptus oil poisoning mentioned by you in your journal of June 2nd was fully commented on by the Australian press at the time of its occurrence.

The case was referred to the Pharmaceutical Society of Tasmania by the Attorney-General for their opinion. The council discussed the subject and passed the following resolution: "That having heard read the depositions which were taken at the inquest held upon the body of Victor Bradshaw, we are of opinion that it is very doubtful whether the child died from poisoning by eucalyptus oil at all; but inasmuch as the case presents certain medical aspects which would be better discussed by the Court of Medical Examiners, we consider the matter should be referred to that body for its opinion."

Mr. Gould, writing from Hobart to the *Chemist and Druggist*, says: "The opinion here was strongly against the theory of poison in the case at all. Most medical men considered it a case of pneumonia which had reached a fatal stage before medical aid was called in. There were no proofs whatever that eucalyptus oil had been taken into the stomach, and beyond the fact that about an inch of the contents of the bottle had gone, no evidence at all of the child having taken it was given. The father said the oil had been sprinkled freely over the room, which would quite account for its disappearance."

Eucalyptus oil is poisonous in large doses as turpentine and all essential oils, but only a few cases have yet occurred which exhibited symptoms of poisoning. Dr. Benjafield, in a letter to the *Lancet*, October 8th, 1892, relates two. One, a child aged two years, took a teaspoonful on sugar. "Within half an hour she began to stagger. Then she became violently sick and lost all power over her legs, so that when attempting to stand she dropped down helpless. A sort of intoxicated torpor then set in. We could awaken her by shaking and calling, but she immediately relapsed. The pulse was quick and strong. This lasted about three hours, when she awoke up perfectly right and ate a good meal. Another child drank an unknown quantity out of a bottle and had the above symptoms very marked; also she had great difficulty in breathing, but she soon recovered."

There was so much doubt about the case of Bradshaw as to whether he took the oil at all, and certainly his pleural cavities contained a quart of serous blood, confirming the general medical opinion in the colony that he died of pneumonia, that I think it unwise to take this case as one of poisoning by eucalyptus oil. A fatal case has been mentioned as having occurred at Geelong, where a man took over an ounce for influenza and died, but no fatal case has as yet been medically reported. J. B. Curgenven.

Teddington, June 6th, 1894

